# Dysregulation of micro-RNA 143-3p as a Biomarker of Carotid Atherosclerosis and the Associated Immune Reactions During Disease Progression

**DOI:** 10.1007/s12265-024-10482-1

**Published:** 2024-01-25

**Authors:** Paula González-López, Yinda Yu, Shiying Lin, Óscar Escribano, Almudena Gómez-Hernández, Anton Gisterå

**Affiliations:** 1 Department of Biochemistry and Molecular Biology, Faculty of Pharmacy, Complutense University of Madrid, Madrid, Spain; 2 Department of Medicine Solna, Center for Molecular Medicine, Karolinska University Hospital, Karolinska Institutet, Stockholm, Sweden; 3 Centro de Investigación Biomédica en Red de Diabetes y Enfermedades Metabólicas Asociadas (CIBERdem), Instituto de Salud Carlos III, Madrid, Spain; 4 Bioclinicum J8:20, Karolinska University Hospital, Visionsgatan 4, Solna, SE-17164 Stockholm, Sweden

**Keywords:** Atherosclerosis, Micro-RNA, Carotid stenosis, Dyslipidemia, T-lymphocytes

## Abstract

Atherosclerosis commonly remains undiagnosed until disease manifestations occur. The disease is associated with dysregulated micro(mi)RNAs, but how this is linked to atherosclerosis-related immune reactions is largely unknown. A mouse model of carotid atherosclerosis, human *APOB100*-transgenic *Ldlr*^−/−^ (HuBL), was used to study the spatiotemporal dysregulation of a set of miRNAs. Middle-aged HuBL mice with established atherosclerosis had decreased levels of miR-143-3p in their carotid arteries. In young HuBL mice, early atherosclerosis was observed in the carotid bifurcation, which had lower levels of miR-15a-5p, miR-143-3p, and miR-199a-3p, and higher levels of miR-155-5p. The dysregulation of these miRNAs was reflected by specific immune responses during atheroprogression. Finally, levels of miR-143-3p were 70.6% lower in extracellular vesicles isolated from the plasma of patients with carotid stenosis compared to healthy controls. Since miR-143-3p levels progressively decrease when transitioning between early and late experimental carotid atherosclerosis, we propose it as a biomarker for atherosclerosis.

**Graphical Abstract:**

Low levels of miR-143-3p in plasma extracellular vesicles can serve as a biomarker for atherosclerosis, and dysregulation of microRNAs is linked to the immune reactions associated with disease progression

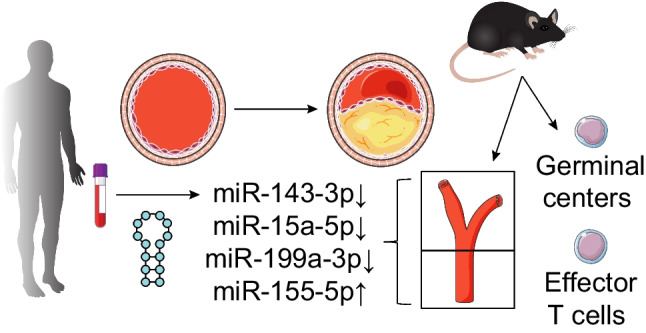

**Supplementary Information:**

The online version contains supplementary material available at 10.1007/s12265-024-10482-1.

## Introduction

Immune-vascular interactions drive the atherosclerotic process, along with hemodynamic turbulence at predilection sites such as the carotid bifurcation [1, 2]. This leads to the buildup of plaque in the carotid arteries, which is a major risk factor for ischemic stroke that causes morbidity and neurological sequelae. As atherosclerosis is increasingly asymptomatic [3], there is a demand for early non-invasive biomarkers to initiate and motivate lifestyle improvements. Early diagnosis and monitoring of treatment responses using biomarkers could become even more important when novel therapeutic approaches are introduced.

The dysregulation of miR-143-3p has been implicated in carotid atherosclerosis and related vascular diseases [4–9]. miR-143-3p is a small, non-coding RNA molecule that plays a crucial role in the regulation of a broad range of target genes, such as *Elk1* mRNA [10]. In individuals with carotid artery plaques, the expression of miR-143-3p is downregulated [6, 8] but conflicting results regarding its effects and regulation exist from experimental models and observational studies [11–15]. Due to proximity, miR-143 is co-transcribed with miR-145, and these are among the highest expressed miRNAs in the medial layer of the vessel wall [4]. Laminar flow induces the expression of miR-143 in endothelial cells and upstream regulators are serum response factor, homeobox protein Nkx-2.5, myocardin, and transforming growth factor-β signaling [10, 16]. miR-143-3p can be packaged and released extracellularly in microvesicles and exert atheroprotective effects in vascular cells or be secreted in the circulation [16]. Intravenous injections of extracellular vesicles containing miR-143-3p may reduce the progression of atherosclerosis in mice [17]. In smooth muscle cells, miR-143-3p represses proliferation and maintains cellular contractility by suppressing transcriptional regulators important for de-differentiation to a synthetic cell state [5, 10]. However, the regulation of miR-143 in immune cells during atheroprogression has not been explored. Further knowledge of this regulation is important since local and systemic shifts in miRNA levels occurring during atheroprogression have broad regulatory consequences.

In this regard, miRNAs have been proposed not only as modulators of the disease but also as potential early- or late-stage biomarkers of atherosclerosis. Therefore, we wanted to study the alteration of miR-15a-5p, miR-143-3p, miR-155-5p, and miR-199a-3p in mouse carotids and immune cells; and as a proof-of-principle, investigate if miR-143-3p is a potential biomarker of late-stage human carotid atherosclerosis. Our previous studies showed that miR-155-5p was upregulated, while miR-15a-5p, miR-143-3p, and miR-199a-3p were downregulated in human and experimental atherosclerosis and have proposed miR-15a-5p and miR-199a-3p as biomarkers of advanced human carotid atherosclerosis [8, 18]. In the present study, we propose the downregulation of miR-143-3p as a potential non-invasive biomarker of the disease. A mouse model of carotid atherosclerosis sheds further light upon the dysregulation of the above-mentioned set of miRNAs in carotid atherosclerosis and their associations with immune reactions. The data indicate that miR-143-3p reflects early atherosclerosis as well as atheroprogression and that its expression is associated with immune processes not limited to the vessel wall.

## Material and Methods

### Human Samples

Plasma samples from healthy donors (n = 13) and patients with advanced carotid atherosclerosis (n = 28) were analyzed. Sample collection and extracellular vesicle isolation were previously described [18], and the use of this cohort was extended by analyzing miR-143-3p levels. Patients were 67 ± 10 years old (see Table S1 for more details).

### Mouse Experiments

Human *APOB100*-transgenic *Ldlr*^tm1Her^ (HuBL) and *Ldlr*^tm1Her^ mice, both on C57BL/6J background, were used. Mice were fed a standard chow diet (2018 Teklad global 18% protein rodent diet, Envigo) and water ad libitum, and were maintained in conventional light, humidity, and heat conditions. Female mice were sacrificed at 11 and 46 weeks of age and male mice were sacrificed at 52 weeks of age. Spectral flow cytometry was performed using fluorophore-conjugated antibodies (Table S2). RNA was analyzed using real-time PCR (Table S3). See the Supplemental Material and Methods for more details.

### Statistics

Data are presented with scatter dot plots with mean and standard deviation. The Mann-Whitney test was used for the comparison of two groups. Data normality was assessed using the Shapiro-Wilk test. Calculations were performed in GraphPad Prism 9.5.1.

## Results

### Decreased miR-143-3p in Advanced Carotid Atherosclerosis

**Fig. 1:**
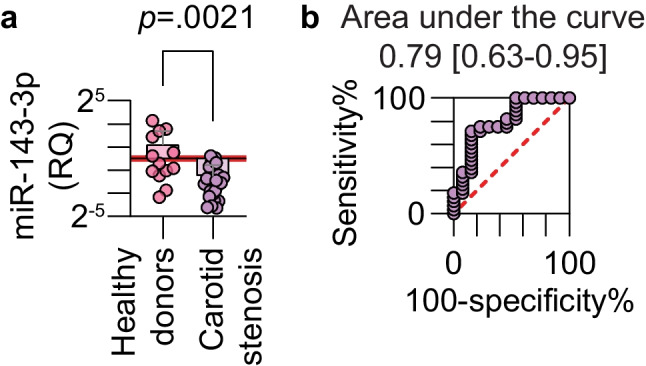
Plasma detection of miR-143-3p in carotid stenosis patients. (**a**) miRNAs in extracellular vesicles were isolated from the plasma of patients with advanced atherosclerosis and the plasma from healthy donors without manifest atherosclerosis. The relative expression of miR-143-3p was measured by real-time PCR (Mann-Whitney test, healthy donors n = 13, carotid stenosis n = 28, log_2_-scaled *y*-axis) (**b**) A receiver operating characteristic curve analysis illustrates how miR-143-3p can be used as a biomarker for carotid atherosclerosis

miRNAs in extracellular vesicles were isolated from plasma samples of patients with advanced carotid atherosclerosis and donors without manifested atherosclerosis to assess the relative levels of miR-143-3p by real-time PCR. Median miR-143-3p levels were 70.6% lower in the plasma of patients with carotid stenosis (Fig. 1a). No significant difference was observed in terms of sex and diabetes (Fig. S1). A receiver operating characteristic curve analysis showed an area under the curve of 0.79 (*p* = 0.0027) and a cut-off value < 0.34 produced a sensitivity of 71.4% and a specificity of 84.6% for advanced disease (Fig. 1b). This confirms that miR-143-3p could be a potential biomarker of carotid stenosis and atherosclerosis in humans.

### Altered miRNAs With Age and Atherosclerosis Severity in Mice

**Fig. 2:**
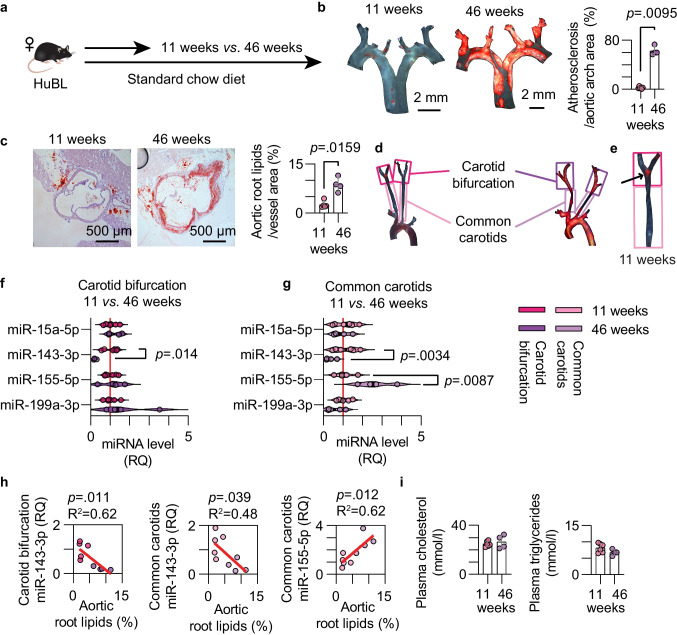
Dysregulation of a set of miRNAs in mouse carotid arteries. (**a**) Experimental setup comparing atheroprogression in HuBL mice. (**b**) Micrographs of Sudan-IV-stained aortic arches with a 2 mm scale bar and quantification of the atherosclerotic plaques (orange color) divided by total aortic arch area. (**c**) Micrographs of Oil Red O-stained sections 300 µm from the aortic root with a 500 µm scale bar. Mean aortic root lipids were quantified (red color) and divided by aortic cross-section area. (**d**) Sudan-IV stained aortic arches with branches to depict microdissection of common carotids and carotid bifurcation. (**e**) Micrograph of a carotid artery from an 11-week-old HuBL mouse, the arrow indicates Sudan-IV^+^ plaque in the carotid bifurcation. (**f-g**) Relative quantification of miRNAs in the carotid bifurcation (bright colors) and common carotids (pale colors) from the 11-week (pink colors, n = 6) and 46-week (purple colors, n = 4) old female HuBL mice. (**h**) Linear regression between lipid deposition in the aortic root and the levels of miR-143-3p in the carotid bifurcation and between lipid deposition in the aortic root and the levels of miR-155-5p and miR-143-3p in the common carotids. (**i**) Plasma concentration of cholesterol and triglycerides

By comparing young (11 weeks) and middle-aged (46 weeks) female HuBL mice we were able to assess atheroprogression (Fig. 2a). The median atherosclerotic burden in the en-face-prepared aortic arches was 28.4 times higher in the 46-week group (Fig. 2b). Oil Red O staining of the aortic root showed 3.9 times higher lipid-stained lesions in the 46-week group (Fig. 2c). Body weight, spleen weight, splenocyte count, and blood leukocytes were not significantly altered with age in this cohort of mice (Table S4).

The carotid bifurcation and common carotids were microdissected as displayed in a set of Sudan-IV-stained arteries (Fig. 2d). Initial lesion formation was observed in the carotid bifurcation at 11 weeks (Fig. 2e). Relative levels of miR-15a-5p, miR-143-3p, miR-155-5p, and miR-199a-3p were assessed but only the expression of miR-143-3p was significantly downregulated in the carotid bifurcation of the middle-aged HuBL mice (Fig. 2f). In the common carotids, miR-155-5p levels were significantly increased with age in addition to the downregulated miR-143-3p (Fig. 2g). Further supporting these changes, carotid miR-143-3p levels showed a negative association with disease progression as measured by Oil Red O staining in the aortic roots, and miR-155-5p levels in the common carotids showed a positive association with disease progression (Fig. 2h). The age difference between the two groups did not significantly impact plasma cholesterol and triglyceride levels (Fig. 2i). This would indicate that the disease process itself, and not hypercholesterolemia, drives these local miRNA changes at predilection sites in the vasculature.

**Fig. 3:**
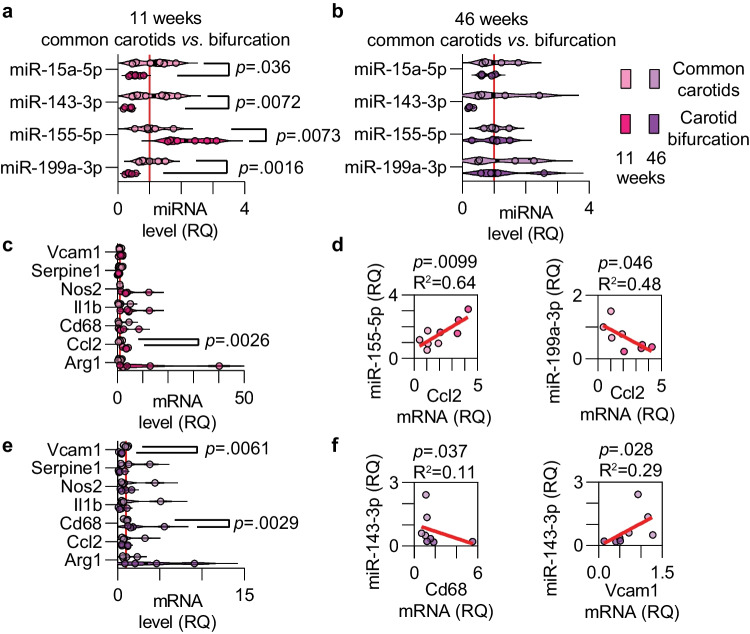
Site-specific miRNA level changes in early and late carotid atherosclerosis. (**a-b**) Relative quantification of miRNAs in the common carotids (pale colors) and carotid bifurcation (bright colors) from the 11-week (pink colors, n = 6) and 46-week (purple colors, n = 4) old female HuBL mice. (**c**) mRNA levels for genes of interest in 11-week-old mice. (**d**) Linear regression between *Ccl2* mRNA and miR-155-5p (left) and miR-199a-3p (right) in carotids with early atherosclerosis. (**e**) mRNA levels for genes of interest in 46-week-old mice. (**f**) Linear regression between *Cd68* mRNA (left) and *Vcam1* mRNA (right), respectively, and miR-143-3p in carotids with advanced atherosclerosis

To assess whether there was site-specific miRNA dysregulation in either early or advanced atherosclerosis, we compared the carotids separately for the 11- and 46-week age groups (Fig. 3a-b). The most striking changes were observed when comparing miRNA levels between the common carotids and the carotid bifurcation in early atherosclerosis: miR-15a-5p, miR-143-3p, and miR-199a-3p were significantly downregulated, and miR-155-5p was upregulated (Fig. 3a). In advanced atherosclerosis, there was no significant difference in miRNA expression between the carotid bifurcation and common carotids (Fig. 3b), likely due to the extension of the disease to the common carotids at this time point.

mRNA levels for a handful of genes related to atherosclerosis progression were investigated in a site-specific manner in the carotids. In early atherosclerosis, only *Ccl2* mRNA, encoding monocyte chemoattractant protein 1, was overexpressed in the carotid bifurcation compared with the common carotid (Fig. 3c). *Ccl2* mRNA showed a strong positive relationship with miR-155-5p expression and was inversely correlated with miR-199a-3p levels (Fig. 3d). In advanced atherosclerosis, *Vcam1* mRNA, encoding vascular cell adhesion molecule 1, was increased in the common carotids and *Cd68*, a highly expressed mRNA in macrophages, was increased in the carotid bifurcation (Fig. 3e), indicating that these sites were in different phases of atheroprogression. *Cd68* mRNA levels were negatively associated with miR-143-3p whereas *Vcam1* mRNA levels were positively associated with miR-143-3p (Fig. 3f). Although these associations were very weak, they support the notion that miR-143-3p is dysregulated in advanced atherosclerosis.

Since there was a site-specific alteration of mRNA levels, we wanted to assess whether the expression was altered by disease severity. When comparing the mRNA levels of the selected genes of interest in the carotid bifurcation of 11 *vs*. 46-week mice, *Nos2* mRNA, encoding inducible nitric oxide synthase, and *Ccl2* mRNA were found to be increased (Fig. S2a). However, these transcript levels did not correlate with any of the measured miRNAs. In the common carotids, there was an increase in mRNA levels of *Vcam1*, *Ccl2*, and *Il1b*, the latter encoding the interleukin-1β cytokine precursor, in middle-aged mice (Fig. S2b). The expression of these three transcripts showed a positive relationship with miR-155-5p levels (Fig. S2c). At the same time, *Ccl2* and *Vcam1* mRNA were inversely correlated with miR-143-3p expression (Fig. S2d). This further denotes the differential expression of miR-143-3p and miR-155-5p in the common carotids, which is a vascular site that typically has laminar blood flow and is affected late in the atherosclerotic process.

### Altered miRNAs in Immune Cells of Atherosclerotic Mice

**Fig. 4:**
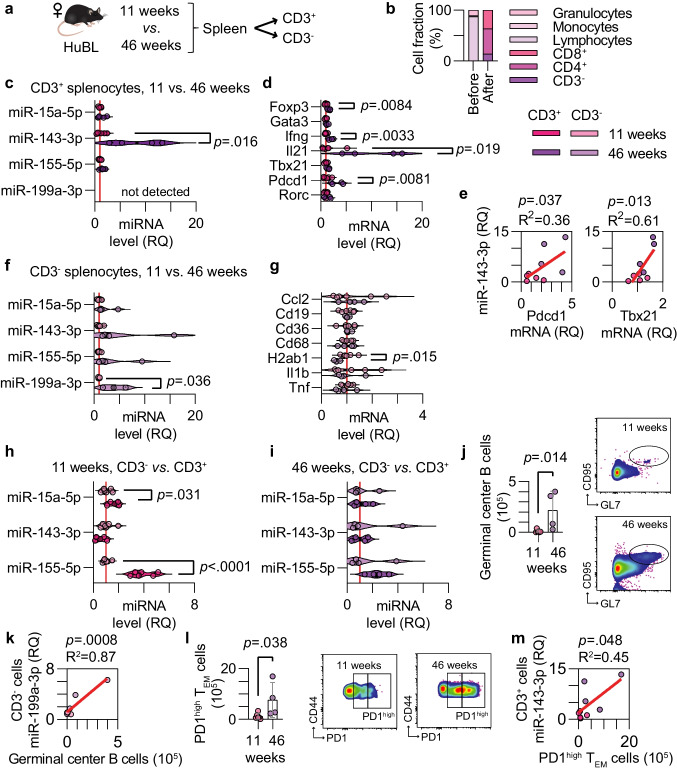
miRNA levels in splenic immune cells during atheroprogression. (**a**) Experimental setup of CD3^+^ and CD3^−^ cell isolation. (**b**) Cell fractions before and after isolation of CD3^+^ cells from the spleen. (**c**) miRNA levels in CD3^+^ cells from the 11-week (pink, n = 6) and 46-week (purple, n = 4) old female HuBL mice. (**d**) mRNA levels for genes of interest in CD3^+^ cells. (**e**) Linear regression between miR-143-3p in CD3^+^ cells and *Pdcd1* and *Tbx21* mRNA. (**f**) miRNA levels in CD3^−^ cells. (**g**) mRNA levels in CD3^−^ cells. (**h-i**) miRNA levels in the CD3^+^ and CD3^−^ cells from the 11- and 46-week groups. (**j-k**) Germinal center B cells as quantified using flow cytometry from the mediastinal and renal lymph nodes and their correlation to miR-199a-3p in splenic CD3^−^ cells. (**l-m**) PD1^high^ T_EM_ cells in the mediastinal and renal lymph nodes and their correlation to miR-143-3p in splenic CD3^+^ cells

We investigated whether the selected miRNAs were altered in immune cells during early and advanced carotid atherosclerosis since previous studies mainly focused on their role in resident vascular cells. CD3^+^ T cells and CD3^−^ non-T cells were isolated from the spleen of young and middle-aged mice (Fig. 4a-b). When studying the miRNA expression in the splenic CD3^+^ T cells, miR-143-3p was selectively increased in mice with advanced atherosclerosis, and miR-199a-3p was non-detectable in those cells (Fig. 4c). A handful of transcripts related to the T-cell phenotype were analyzed. In mice with advanced atherosclerosis, *Foxp3*, *Ifng*, *Il21*, and *Pdcd1* mRNA levels were increased in the splenic CD3^+^ cells indicating that atheroprogression drives T-cell activation and regulatory transcriptional programs (Fig. 4d). *Pdcd1* mRNA, encoding the immune checkpoint programmed cell death protein 1 (PD1), and *Tbx21* mRNA, encoding the T-box transcription factor expressed in T-helper type 1 cells, were positively correlated with miR-143-3p expression in those cells, implicating this miRNA in the context of immune regulation (Fig. 4e).

When analyzing the relative expression of miRNAs in the CD3^−^ cells, only miR-199a-3p was increased during atheroprogression (Fig. 4f). A few transcripts involved in inflammatory pathways were analyzed, and mRNA from the MHC class II encoding gene *H2ab1* was found to be significantly decreased in the non-T cell fraction (Fig. 4g), indicating that antigen presentation could be differentially regulated during atheroprogression in the spleen. To observe specific enrichments in the immune cell compartment during atheroprogression, we compared if there was an alteration in miRNAs between splenic CD3^−^ and CD3^+^ cells in early and advanced atherosclerosis. In early atherosclerosis, significant enrichments of miR-15a-5p and miR-155-5p were found in CD3^+^ cells (Fig. 4h), while this enrichment was subdued in advanced atherosclerosis (Fig. 4i).

Specifically, cellularity in the aorta-associated mediastinal and renal lymph nodes was increased, while cellularity remained at a similar level in the spleen (Fig. S3a-b and Table S4). An expansion of germinal center B cells and plasma cells was observed in the mediastinal and renal lymph nodes as analyzed by flow cytometry (Fig. 4j and S3c-f). The expansion of plasma cells was, at this time point and location, limited to the absolute cell counts (Fig. S3d-e). As miR-199a-3p is known to be upregulated in germinal center B cells [19], this miRNA correlated with the germinal center reactions (Fig. 4k, Fig. S3n).

Blood lymphocytes as well as total T cell number in the mediastinal and renal lymph nodes were not significantly different between groups (Fig. S3g-h and Table S4). However, there was an increase in the total and relative number of T effector/memory (T_EM_) cells (Fig. S3i-j). Confirming the upregulated *Pdcd1* mRNA levels, PD1^high^ T_EM_ cells were found to accumulate in the aorta-associated lymph nodes during atheroprogression (Fig. 4l and S3l). Speculatively, persistent presentation of atherosclerosis-associated antigens may be driving these effects. The T_EM_ cell with intermediate PD1 expression remained unaltered (Fig. S3k-m). Expression of miR-143-3p in T cells correlated with the number of PD1^high^ T_EM_ cells (Fig. 4m). Since the correlation to *Tbx21* mRNA was stronger, this might reflect an upregulation in activated T cells or Th1 cells more specifically. However, this could not be distinguished using our experimental design.

### Altered miRNAs in Middle-aged Mice

**Fig. 5:**
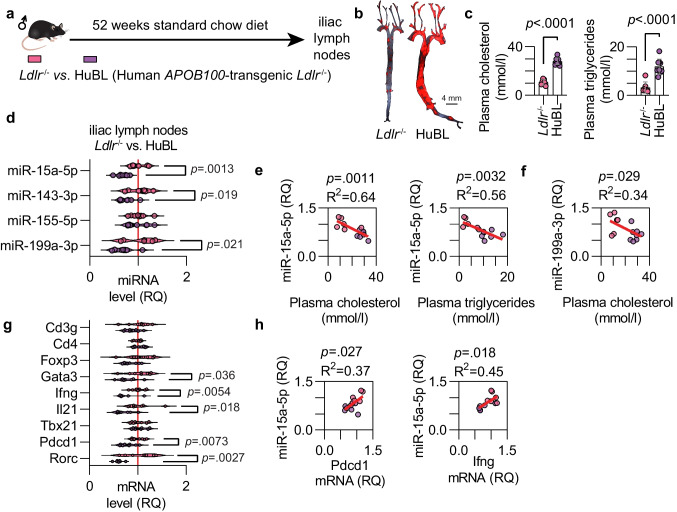
Dysregulated transcripts in iliac lymph nodes of middle-aged mice. (**a**) Experimental setup. (**b**) Micrographs of Sudan-IV-stained aortas with a 4 mm scale bar. (**c**) Total plasma cholesterol and triglycerides concentrations. (**d**) Relative quantification of miRNAs in the iliac lymph nodes from male *Ldlr*^*−/*−^ (pink color, n = 8) and HuBL mice (purple color, n = 8). (**e–f**) Linear regression between miR-15a-5p and miR-199-3p with plasma cholesterol and triglycerides. (**g**) mRNA levels for genes of interest in the iliac lymph nodes. (**h**) Linear regression between *Pdcd1* and *Ifng* mRNA, respectively, and miR-15a-5p levels in the iliac lymph nodes

Finally, we investigated whether the dysregulation of miRNAs occurs during atheroprogression without the influence of age differences. Age-matched one-year-old male *Ldlr*^*−/−*^ and HuBL mice fed a standard chow diet were used for this purpose (Fig. 5a). The atherosclerotic burden was 7.0-fold increased in the aorta of HuBL mice as driven by more severe hypercholesterolemia induced by transgenic overproduction of *APOB100* (Fig. 5b-c). No difference in body weight was recorded but HuBL mice had higher blood counts of lymphocytes, monocytes, and granulocytes (Table S5) as well as plasma cholesterol and triglyceride levels (Fig. 5c). To analyze miRNA levels, the iliac lymph nodes in proximity to the aorta were used. Levels of miR-15a-5p, miR-143-3p, and miR-199a-3p were lower in HuBL compared to *Ldlr*^*−/−*^ mice (Fig. 5d). This is similar to our findings in the vasculature of female HuBL mice at different ages but in the iliac lymph nodes of those mice, only miR-143-3p was found to be significantly decreased (Fig. S4).

miR-15a-5p showed a negative association with plasma lipid levels, indicating its inverse association with hypercholesterolemia (Fig. 5e). A weaker association was observed between miR-199a-3p and plasma cholesterol (Fig. 5f). miR-143-3p did not correlate with hypercholesterolemia levels, indicating that its dysregulation is more associated with disease progression than dyslipidemia. However, atheroprogression is tightly linked to hypercholesterolemia in these mice, and separating these entities is challenging.

A few selected transcripts related to T-cell phenotype were analyzed. *Gata3* mRNA, encoding a transcription factor involved in T-helper type 2 responses, *Ifng* mRNA, encoding interferon-γ, *Il21* mRNA, encoding the T follicular helper-related cytokine interleukin-21, *Rorc* mRNA, encoding retinoic acid receptor-related orphan receptor C associated with T-helper 17 cells, and *Pdcd1* mRNA were all observed to be lower in the HuBL mice (Fig. 5g). *Pdcd1* and *Ifng* correlated with miR-15a-5p expression (Fig. 5h). Taken together, severe dyslipidemia was identified as a contributing mediator of dysregulation of miR-15a-5p and, to a lesser extent, miR-143-3p.

## Discussion

We show that the dysregulation of miR-143-3p in carotid atherosclerosis reflects the progression of the disease and that it potentially could be used as a screening tool for atherosclerosis or for non-invasive disease staging. In our mouse model, we found that miR-143-3p was decreased in early and advanced atherosclerosis, as well as in the carotid bifurcation and common carotids. Downregulation of miR-143-3p has been observed in both human carotid plaques and animal models of atherosclerosis before [8]. The novel aspect of this study is the mapping of the dysregulation of miR-143-3p to different parts of the carotid arteries in mice, and that we show an upregulation of miR-143-3p in T cells during atheroprogression. Notably, miR-143-3p is transmitted by the endothelium to the circulation in extracellular vesicles and to vascular smooth muscle cells to keep them in a contractile state. T cells also release extracellular vesicles constitutively [20], which opens the possibility that T cells can communicate with and affect gene expression in vascular resident cells through miR-143-3p. However, whether miR-143-3p is a cargo of extracellular vesicles from T cells and whether they could reach vascular smooth muscle cells remains to be investigated.

The differential regulation of miR-143-3p between the vasculature and the immune cell compartment might explain some conflicting observations in the field [12–15]. miR-143-3p downregulation has previously been observed in *Apoe*^−/−^ and *Ldlr*^−/−^ mouse aortas when comparing mice on a high-fat diet with controls fed a chow diet [8, 21]. This finding is corroborated by our data showing that miR-143-3p is continuously decreased with age and atheroprogression. We therefore propose that it could be used as a marker for atheroprogression. Experiments of balloon-injured carotid arteries in rats provide further support for this notion [22]. However, several miRNAs are downregulated in atherosclerosis and further comparisons are needed to select the most selective and sensitive markers for vascular dysfunction. PCR-based extracellular vesicle testing is not a clinically convenient methodology but can be multiplexed, adding together a set of markers to increase specificity.

miRNAs exert their effects through binding complementary sequences in mRNA molecules and, due to their versatile effects, are not unanimously considered promising novel therapeutic targets anymore. Our bulk tissue analysis of carotids found miR-143-3p to be associated with *Cd68*, *Vcam1*, and *Ccl2* mRNA. miR-143-3p is not known to bind those targets directly but has been shown to be an indirect modulator of *Vcam1* [23] and *Ccl2* [24] mRNA. Its effects are challenging to distinguish from the cotranscribed miR-145 and *Carmn* non-coding RNA, which are in proximity to the miR-143 locus. However, detailed mapping of this intricate coregulatory network is not critical if miR-143-3p is viewed as a strict biomarker.

Immune-vascular interactions are drivers of atherosclerosis progression but how miRNAs are involved in these processes is less explored. We made a simple comparison of miRNA levels in splenic T cells and non-T cells during atheroprogression. Surprisingly, this revealed that miR-143-3p was overexpressed in T cells in mice with advanced atherosclerosis. To further characterize the T cells, we studied genes related to their phenotype, showing an increase in mRNA levels of *Il21*, *Ifng*, *Foxp3*, and *Pdcd1*. Interestingly, only *Pdcd1*, encoding PD1, correlated with miR-143-3p. Additionally, this miRNA also correlated with the PD1^high^ T-helper cells found in the aorta-associated lymph nodes. PD1 is highly expressed on activated and exhausted T cells and is commonly targeted with immune checkpoint inhibitors in cancer treatment to increase T-cell reactivity. Blocking PD1 could at the same time increase the production of pro-atherogenic cytokines in iliac lymph nodes [25]. One prior report corroborates our finding that miR-143-3p is expressed in T cells [26]. Wang et al. studied cytokine-induced killer cells, which include cytotoxic T cells, and report that miR-143-3p is involved in regulating T-cell activation and proliferation. Whether miR-143-3p is involved in T-cell activation in our model and how this is related to pathological processes in the vascular wall remains to be investigated.

Another miRNA we measured in our atheroprogression model was miR-15a-5p, which is widely studied in the cancer field. Tumor tissue often has downregulated miR-15a-5p levels, which leads to less regulated gene expression that, in turn, could facilitate tumor growth and carcinogenesis [27]. In atherosclerosis, we found a similar effect with downregulated miR-15a-5p in early atherogenesis and iliac lymph nodes in older mice. A few reports have studied the mechanistic implications of miR-15a-5p in a vascular context and have shown that it could reduce inflammatory changes in the endothelium [28] and promote smooth muscle cell migration [29], i.e., effects that could be interpreted as beneficial for atherosclerosis and plaque stability, respectively. We found that miR-15a-5p levels were higher in splenic T cells compared to non-T cells during early atherogenesis. With disease progression, this difference was eliminated. miR-15a-5p has been associated with activated cytotoxic T cells in a tumor microenvironment [30] and its downregulation could boost T-helper cell responses in asthmatic airways [31]. Moreover, the expression of miR-15a-5p in B cells has been associated with vaccination responses [32]. We did not observe a significant difference in miR-15a-5p levels in splenic cells that could indicate a similar effect during atheroprogression. As relative miRNA levels were measured, further controls are required to understand whether levels increased or decreased in certain lymphocyte subsets. Nonetheless, the observed lower levels in the iliac lymph nodes of HuBL mice could implicate its involvement in reduced cellular antigen responsiveness during hypercholesterolemia. Moreover, in the age-matched mice with more advanced atherosclerosis due to severe dyslipidemia, miR-15a-5p correlated positively with *Pdcd1* and *Ifng* mRNA and inversely with plasma cholesterol and triglyceride levels. *Pdcd1* mRNA is a confirmed target of miR-15a-5p [33], and our data are seemingly at odds with this effect. However, the positive association with *Ifng* mRNA was stronger according to our data, and interferon-γ is an upstream modulator that increases miR-15a-5p expression [34]. Taken together, our study design is more suited to detect commonly regulated pathways in atherosclerosis and not to detect miRNA-targeted effects. Mechanistic studies are needed to unravel how dyslipidemia could cause dysregulation of miR-15a-5p and whether it is mediated through interferon signaling.

miR-155-5p is, in a cardiovascular context, the most studied of the four miRNAs we measured. Previous reports have found it to be both increased [35] and decreased [36] in plasma samples from patients with advanced atherosclerosis. Functionally, it has been associated with increased cholesterol efflux from macrophages and reverse cholesterol transport [37, 38]. In our mouse model, we found that miR-155-5p was increased in carotid bifurcations with early atherosclerosis and in common carotids during disease progression. In the latter, it correlated positively with *Ccl2*, *Vcam1*, and *Il1b* mRNA. These mRNAs are not predicted to be targets of miR-155-5p but are under transcriptional control by the nuclear factor-κB pathway [39], which is activated in atherosclerosis. Levels of miR-155-5p were higher in splenic CD3^+^ cells compared to CD3^−^ cells in mice with initial atherosclerosis. In the literature, miR-155-5p has been studied as an exosomal signal from different types of T cells, *e.g*., miR-155-5p could increase activation of cytotoxic T cells, which decreases tumor growth in an ovarian cancer model [40]. Possibly, a similar effect on T cells would play only a minor role in atheroprogression since the miR-155-5p elevation in T cells was not observed in middle-aged mice with more pronounced disease.

Finally, we found that miR-199a-3p is downregulated in early carotid atherosclerosis in mice. A similar downregulation of miR-199a-3p was observed in iliac lymph nodes in 1-year-old HuBL mice compared to *Ldlr*^−/−^ mice. Previously, miR-199a-3p has been reported to be involved in endothelial dysfunction [41] and the inhibition of vascular smooth muscle cell migration [42]. The most striking data for this miRNA in our study was the upregulation in non-T splenocytes and its association with germinal center B-cell responses. During atheroprogression, germinal centers expand in the aorta-associated lymph nodes. Such reactions are central to the generation of affinity-matured B cells in response to T-dependent antigens, leading to the maturation of long-lived plasma cells, the generation of memory B cells, and the production of high-affinity antibodies. Our results are consistent with previous studies focusing on miRNAs in B-cell responses and germinal centers [19, 32]. Supporting our observation, we found that splenic miR-199a-3p levels correlate with PD1^high^ effector T cells, which could interact with germinal center B cells.

There are limitations to the present study. It is a translational research study with a small sample size and large effect sizes. Biologically relevant effects of smaller magnitude and sex-specific effects could be missed. The findings are mainly correlative since it is a biomarker study, but as discussed, have generated hypotheses that could be explored mechanistically. The broadly targeted effects of miRNAs limit their therapeutic use but do not influence their potential as biomarkers. Replication of the results in other cohorts has partly been performed previously [4–6, 8, 9]. However, larger sample sizes with matched controls and adjustments for confounders would be needed, and the validation of miR-143-3p dysregulation as a biomarker for early atherosclerosis in humans is still pending. The development of extracellular vesicle extraction coupled with real-time PCR as diagnostic tests would also be required to enable clinical use.

In conclusion, the downregulation of miR-143-3p is a sensitive marker for carotid atherosclerosis in mice, and it has the potential to serve as a non-invasive biomarker for atherosclerosis in humans when measured in plasma extracellular vesicles. Furthermore, since miR-143-3p levels progressively decrease in experimental carotid atherosclerosis, it could be utilized to grade the severity of the disease.

## Supplementary Information

Below is the link to the electronic supplementary material.

Supplementary file1 (PDF 836 KB)

## Data Availability

The data from this study are available from the corresponding author upon reasonable request.

## Abbreviations

HuBL: Human *APOB100*-transgenic *Ldlr*^−^^/^^−^
miRNA: Micro ribonucleic acids
PD1: Programmed cell death protein 1
RQ: Relative quantification
T_EM_ cells: T effector/memory cells
