# Supplementary material for: Dysregulation of micro-RNA 143-3p as a Biomarker of Carotid Atherosclerosis and the Associated Immune Reactions During Disease Progression
Source: J Cardiovasc Transl Res. 2024 Jan 25;17(4):768–78. doi: 10.1007/s12265-024-10482-1 (PMC11371874; doi:10.1007/s12265-024-10482-1)
Supplement: Supplementary file 1 — Supplementary file1 (PDF 836 KB) [file 12265_2024_10482_MOESM1_ESM.pdf]

SUPPLEMENTARY INFORMATION

# Dysregulation of micro-RNA 143-3p as a biomarker of carotid atherosclerosis and the associated immune reactions during disease progression

Paula González-López, Yinda Yu, Shiying Lin, Óscar Escribano, Almudena Gómez-Hernández, Anton Gisterå

*Supplemental Material and Methods*

Mouse experiments

Cell and plasma analyses

RNA isolation, reverse transcription, and quantitative polymerase chain reaction

*Supplemental Figures*

Figure S1. Detection of miR-143-3p in extracellular vesicles stratified according to sex and diabetes.

Figure S2. Site-specific mRNA level alterations in carotids during atheroprogression.

Figure S3. Flow cytometric phenotyping of aorta-associated lymph nodes.

Figure S4. Dysregulation of miR-143-3p in iliac lymph nodes during atheroprogression.

*Supplemental Tables*

Table S1. Basic characteristics of patients with advanced carotid atherosclerosis.

Table S2. List of antibodies used for flow cytometry analysis.

Table S3. List of assay-on-demand used for real-time PCR.

Table S4. Basic characteristics of female HuBL mice.

Table S5. Basic characteristics of 52-week-old male mice.

*Supplemental References*

**Supplemental Material and Methods**

## *Mouse experiments*

The human *APOB100*-transgenic *Ldlr*$^{tm1Her}$ (HuBL, European mutant mouse archive 09689) strain carries the full-length human *APOB100* gene, in which codon 2153 has been converted from glutamine to leucine to prevent the formation of apolipoprotein B48, thus generating only apolipoprotein B100 [1, 2]. From the same in-house breeding colony, *Ldlr*$^{tm1Her}$ mice (Jax strain 002207) lacking the human *APOB100*-transgene, were used for comparing HuBL mice to a model of milder hypercholesterolemia. The mice were euthanized by a $CO_2$ overdose and blood was collected by cardiac puncture in ethylenediaminetetraacetic acid-coated tubes. The vasculature was perfused with ice-cold sodium chloride (9 mg/ml) before the organs were collected. The aortic arch was fixed in buffered 4% formaldehyde solution for later pinning and staining with Sudan IV (Sigma-Aldrich), as previously described [3]. Microdissected carotid arteries were stored in RNAlater reagent (Thermo Fisher) for RNA isolation. The spleen and iliac lymph nodes were snap-frozen for RNA isolation. The heart was preserved in optimal cutting temperature compound and the proximal part of the aortic root was sectioned using a CryoStar NX50 cryostat (Thermo Fisher). Five μm thick sections were fixed in buffered 4% formaldehyde solution and stained with 0.5% Oil Red O (Sigma-Aldrich). Hematoxylin was used to counterstain the nuclei of the cells. Then, the tissues were mounted with an aqueous mounting medium, and micrographs were acquired using a light microscope (Leica DM LB2). The mediastinal and renal lymph nodes were collected in phosphate-buffered saline with 0.1% bovine serum albumin and 1 mM ethylenediaminetetraacetic acid for flow cytometry.

### *Cell and plasma analyses*

Single-cell suspensions were prepared from spleens and $CD3^+$ T cells isolated by negative selection with antibodies to CD11b, CD16/32, CD45R, and Ter-119 (Dynabeads untouched mouse T cells kit, Invitrogen). Flow cytometry was performed using a fixable near-infrared dead cell stain kit (Thermo Fisher). After Fc-block (anti-CD16/32, BD Biosciences), fluorophore-labeled primary antibodies were

used for extracellular staining (Table S2). Samples were acquired on a Cytek Northern Lights 3000 spectral flow cytometer and data were analyzed using FlowJo software (Tree Star). Whole blood, splenocyte, and lymph node single-cell suspensions were analyzed on a Vet animal blood counter (Scil). Plasma cholesterol and triglycerides were analyzed using enzymatic colorimetric kits (Randox).

*RNA isolation, reverse transcription, and quantitative polymerase chain reaction*

The small non-coding miRNAs and the coding messenger RNAs were isolated from plasma extracellular vesicles, mouse carotids, lymph nodes, and cells using the mirVana miRNA isolation kit with phenol (Thermo Fisher), following the manufacturer's protocol and adapting the volumes of the lysis binding buffer for each sample: 100 µL for the carotids, lymph nodes, and splenic $CD3^+$ cells, and 300 µL for splenic $CD3^-$ cells. Total RNA quality was analyzed on a BioAnalyzer instrument (Agilent Technologies) and quantified by 260 nm absorbance using a Nanodrop 1000 spectrophotometer (Thermo Fisher).

Reverse transcription was performed with a high-capacity cDNA reverse transcription kit for the mRNA and using the TaqMan advanced miRNA cDNA synthesis kit for the miRNA. The amplification of the cDNA was performed by real-time PCR using TaqMan universal master mix and pre-manufactured primers and probes (assay-on-demand) for the genes of interest and *Hprt* mRNA, miR-191-5p, and miR-16-5p as internal controls, in a QuantStudio 7 Pro real-time PCR System using the QuantStudio Design & Analysis Software 2.6.0 (all from Applied Biosystems). Data were analyzed using the relative abundance of mRNA or miRNA targets, normalized with the endogenous gene and relative to the control, calculated as follows: Relative quantification (RQ) = $2^{-\Delta\Delta Ct}$; ΔCt (cycle threshold) = Ct (miRNA target) − Ct (endogenous control); ΔΔCt = [ΔCt (for sample) − ΔCt (for the control group)]. Amplification of internal controls was performed simultaneously with all samples.

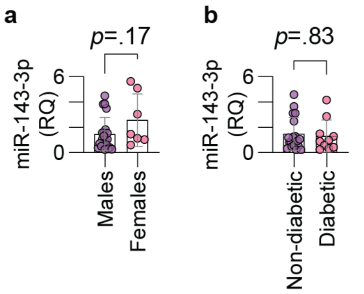


**Fig. S1** Detection of miR-143-3p in extracellular vesicles stratified according to sex and diabetes. **(a)** miRNAs in extracellular vesicles were isolated from the plasma of patients with advanced atherosclerosis. Levels of miR-143-3p were quantified by real-time PCR and stratified according to sex (Mann-Whitney test, males n=21, females n=7) **(b)** Levels of miR-143-3p stratified according to type 2 diabetes mellitus (Mann-Whitney test, non-diabetic n=18, diabetic n=10).

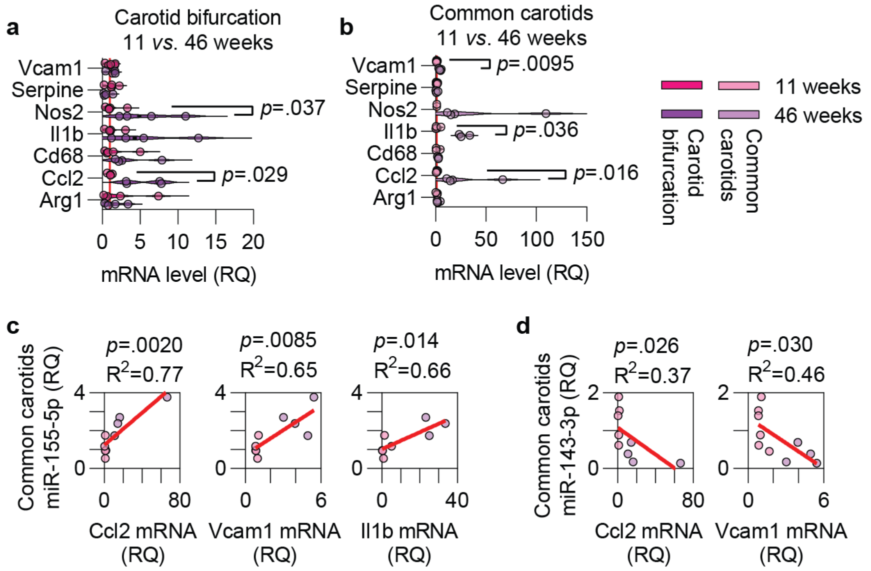


**Fig. S2** Site-specific mRNA level alterations in carotids during atheroprogression. **(a-b)** mRNA levels for genes of interest in the carotid bifurcation and common carotid from 11- and 46-week-old mice. **(c)** Linear regression between *Ccl2*, *Vcam1*, and *Il1b* mRNAs, respectively, and miR-155-5p levels in the common carotids of 11-week-old mice. **(d)** Linear regression between *Ccl2* and *Vcam1* mRNAs, respectively, and miR-143-3p expression in the common carotids of 46-week-old mice. Female HuBL mice, 11 weeks n=6, 46 weeks n=4, RQ=relative quantification.

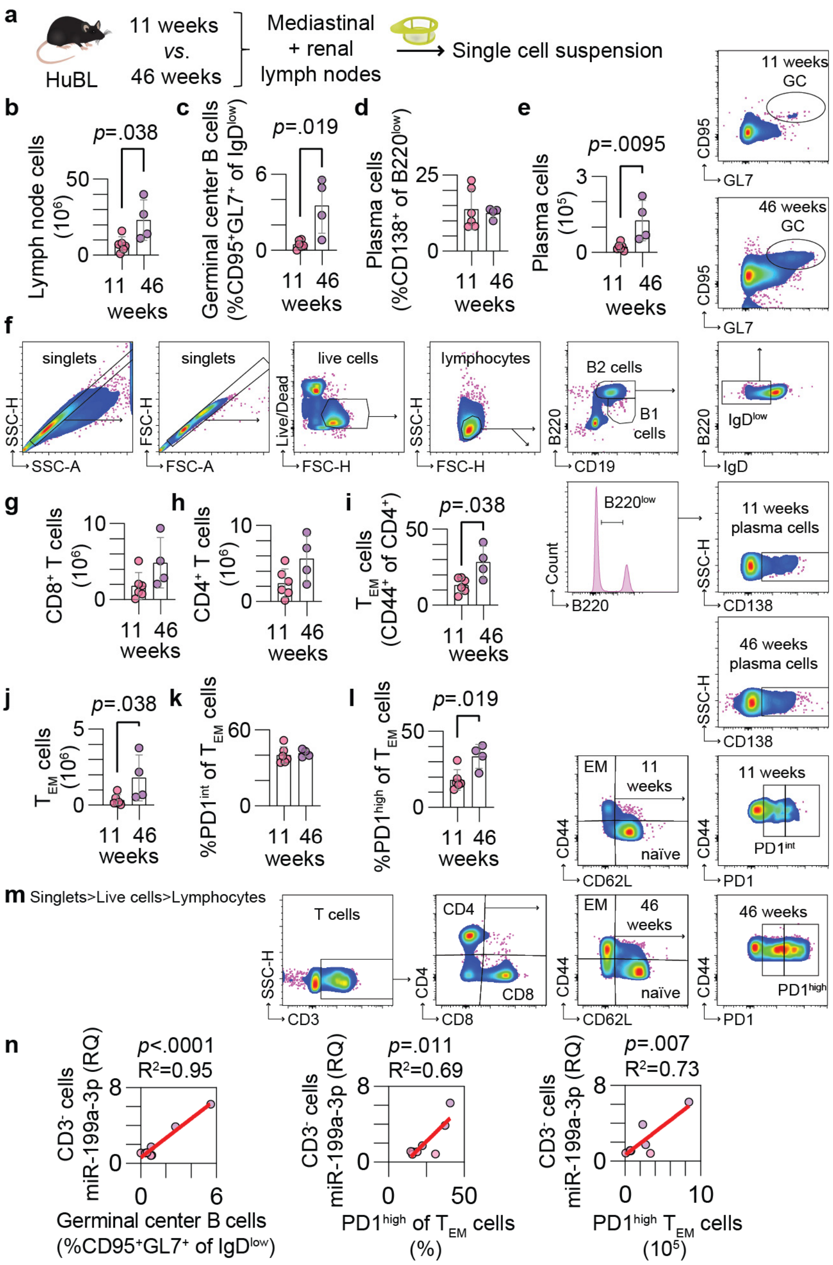


**Fig. S3** Flow cytometric phenotyping of aorta-associated lymph nodes. **(a)** Schematic overview of the experimental setup. **(b)** Cell counts in the pooled mediastinal and renal lymph nodes. **(c)** Relative germinal center B cell frequencies. **(d-e)** Relative fraction and absolute number of plasma cells. **(f)** Gating strategy for flow cytometry analysis of B cells. **(g-h)** Number of $CD8^+$ and $CD4^+$ T cells. **(i)** Relative frequency of $CD44^+CD62L^-$ $T_{EM}$ cells among $CD4^+$ T-helper cells. **(j)** Number of $CD4^+CD44^+CD62L^-$ $T_{EM}$ cells. **(k-l)** Frequency of $T_{EM}$ cells with intermediate and high PD1 expression, respectively. **(m)** Gating strategy for flow cytometry analysis of T cells. **(n)** Linear regression between miR-199-3p in splenic $CD3^-$ cells and germinal center B cell frequency, $PD1^{high}$ $T_{EM}$ cell frequency, and $PD1^{high}$ effector $T_{EM}$ cell number. Female human *APOB100*-transgenic *Ldlr*$^{-/-}$ (HuBL) mice, 11 weeks n=6, 46 weeks n=4, GC=germinal center, EM=effector/memory, PD1=programmed cell death 1, int=intermediate.

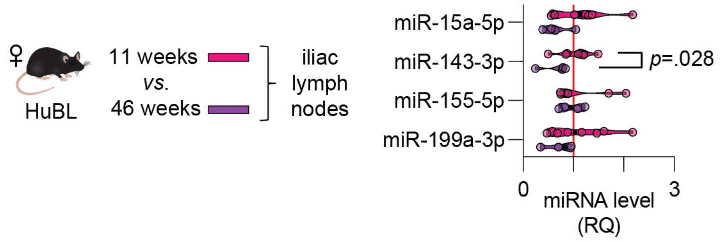


**Fig. S4.** Dysregulation of miR-143-3p in iliac lymph nodes during atheroprogression. Relative quantification (RQ) of miRNAs in the iliac lymph nodes from the 11-week (pink colors, n=8) and 46-week (purple colors, n=4) old female HuBL mice.

**Table S1.** Basic characteristics of patients with advanced carotid atherosclerosis.

| **Characteristic** | **n=28** |
|---|---|
| **Age** (years) | 67 ±10 |
| **Male sex** (%) | 75% |
| **Body weight** (kg) | 79.4 ±13.4 |
| **Body mass index** ($kg/m^2$) | 28.8 ±4.8 |
| **Diabetes mellitus type 2** (%) | 35.7% |
| **Hypertension** (%) | 75% |
| **Current smoker** (%) | 28.6% |
| **Plasma cholesterol** (mmol/l) | 3.95 ±1.30 |
| **Plasma LDL** (mmol/l) | 2.41 ±1.13 |
| **Plasma HDL** (mmol/l) | 1.12 ±0.43 |
| **Plasma triglycerides** (mmol/l) | 1.55 ±1.75 |

**Table S2.** List of antibodies used for flow cytometry analysis.

| Target | Clone | Company | Conjugation | Staining concentration |
|---|---|---|---|---|
| **B220** | RA3-6B2 | BioLegend | BV510 | 0.4 µg/ml |
| **CD3ε** | 145-2C11 | BioLegend | BV510 | 1 µg/ml |
| **CD4** | RM4.5 | BioLegend | BV605 | 0.2 µg/ml |
| **CD8a** | 53-6.7 | BD Biosciences | BV750 | 0.2 µg/ml |
| **CD19** | 1D3 | BD Biosciences | AF700 | 1 µg/ml |
| **CD44** | IM7 | BioLegend | PerCP | 1 µg/ml |
| **CD62L** | MEL-14 | BioLegend | BV650 | 0.4 µg/ml |
| **CD95** | Jo2 | BD Biosciences | BV750 | 1 µg/ml |
| **CD138** | 281-2 | BD Biosciences | BV650 | 1 µg/ml |
| **GL7** | GL7 | BioLegend | AF647 | 1 µg/ml |
| **IgD** | 11-26c.2a | BioLegend | PerCP | 1 µg/ml |
| **PD1** | RMP1-30 | BD Biosciences | BV421 | 1 µg/ml |

**Table S3.** List of assay-on-demand used for real-time PCR.

| Target | Reference number |
|---|---|
| mmu-miR-15a-5p | mmu482962_mir |
| hsa-miR-199a-3p | 477961_mir |
| mmu-miR-155-5p | mmu480953_mir |
| hsa-miR-143-3p | 477912_mir |
| mmu-miR-191-5p | mmu481584_mir |
| mmu-miR-16-5p | mmu482960_mir |
| Hprt | Mm03024075_m1 |
| Vcam1 | Mm01320970_m1 |
| Serpine1 | Mm00435858_m1 |
| Nos2 | Mm00440502_m1 |
| Il1b | Mm00434228_m1 |
| Cd68 | Mm03047343_m1 |
| Ccl2 | Mm00441242_m1 |
| Arg1 | Mm00475988_m1 |
| Foxp3 | Mm00475162_m1 |
| Gata3 | Mm00484683_m1 |
| Ifng | Mm01168134_m1 |
| Il21 | Mm00517640_m1 |
| Tbx21 | Mm00450960_m1 |
| Pdcd1 | Mm01285676_m1 |
| Rorc | Mm01261022_m1 |
| Cd19 | Mm00515420_m1 |
| Cd36 | Mm00432403_m1 |
| H2ab1 | Mm00439216_m1 |
| Tnf | Mm00443258_m1 |
| Cd3g | Mm00438095_m1 |
| Cd4 | Mm00442754_m1 |

**Table S4.** Basic characteristics of female HuBL mice.

| | **11 weeks** n=6 | **46 weeks** n=4 | ***p*-value** |
|---|---|---|---|
| **Body weight** (g) | 21.9 ±0.9 | 24.4 ±2.2 | n.s. |
| **Spleen weight** (g) | 86.8 ±9.4 | 72.3 ±17.4 | n.s. |
| **Splenocytes** ($10^6$) | 164.7 ±29.9 | 154.0 ±17.3 | n.s. |
| **Blood Leukocytes** ($10^9$/l) | 12.2 ±3.1 | 16.9 ±10.4 | n.s. |
| **Blood Lymphocytes** ($10^9$/l) | 9.3 ±2.5 | 10.6 ±7.5 | n.s. |
| **Blood Monocytes** ($10^9$/l) | 0.5 ±0.1 | 0.9 ±0.5 | n.s. |
| **Blood Granulocytes** ($10^9$/l) | 2.4 ±0.6 | 5.4 ±2.9 | n.s. |
| **Mediastinal and renal lymph node cells** ($10^6$) | 7.0 ±5.1 | 23.0 ±13.3 | .038 |

n.s.=not significant

**Table S5.** Basic characteristics of 52-week-old male mice.

| | *Ldlr*$^{-/-}$ | HuBL | *p*-value |
|---|---|---|---|
| **Body weight** (g) | 31.0 ±5.6 n=7 | 30.3 ±1.6 n=9 | n.s. |
| **Blood Leukocytes** ($10^9$/l) | 8.0 ±1.0 n=7 | 11.9 ±1.5 n=9 | .00002 |
| **Blood Lymphocytes** ($10^9$/l) | 5.8 ±0.9 n=7 | 7.8 ±1.2 n=9 | .002 |
| **Blood Monocytes** ($10^9$/l) | 0.5 ±0.1 n=7 | 0.7 ±0.2 n=9 | .02 |
| **Blood Granulocytes** ($10^9$/l) | 1.7 ±0.5 n=7 | 3.4 ±0.7 n=9 | .0001 |
| **Aorta atherosclerosis** (%, en-face-Sudan-IV-stained lesions/aorta area) | 9.1 ±3.2 n=3 | 63.2 ±15.6 n=3 | .02 |

n.s.=not significant

## Supplemental References

1. Skålén K, Gustafsson M, Rydberg EK, Hultén LM, Wiklund O, Innerarity TL, et al. Subendothelial retention of atherogenic lipoproteins in early atherosclerosis. Nature. 2002;417(6890):750-4. doi: 10.1038/nature00804.
2. Gisterå A, Ketelhuth DFJ, Malin SG, Hansson GK. Animal Models of Atherosclerosis-Supportive Notes and Tricks of the Trade. Circ Res. 2022;130(12):1869-87. doi: 10.1161/circresaha.122.320263.
3. Centa M, Ketelhuth DFJ, Malin S, Gisterå A. Quantification of Atherosclerosis in Mice. J Vis Exp. 2019(148). doi: 10.3791/59828.
